# Transmission and Evolution of Antibiotic Resistance Genes and Antibiotic-Resistant Bacteria in Animals, Food, Humans and the Environment

**DOI:** 10.3390/microorganisms14030634

**Published:** 2026-03-11

**Authors:** Linjuan Li, Jie Zhu, Yuxin Yan, Zhangheng Li, Hong Du

**Affiliations:** Department of Clinical Laboratory, The Second Affiliated Hospital of Soochow University, Suzhou 215000, China

**Keywords:** antibiotic resistance genes, antibiotic-resistant bacteria, One Health, transmission, evolution

## Abstract

Antimicrobial resistance (AMR) constitutes one of the most severe and pressing threats to global public health, food security, and environmental integrity. This review synthesizes current evidence across interconnected One Health domains—humans, animals, food, and the environment—to delineate the scope, mechanisms, and drivers of AMR transmission. Our analysis reveals three principal findings. First, the scope of AMR is alarmingly extensive, with antibiotic-resistant bacteria (ARB) and genes (ARGs) now pervasive across all four ecological compartments, transcending traditional clinical boundaries. Second, this widespread distribution is critically facilitated by horizontal gene transfer mechanisms, particularly via mobile genetic elements such as plasmids, which enable ARGs to disseminate rapidly between diverse bacterial populations across different ecosystems. Third, we identify multiple interconnected drivers that actively promote this cross-ecosystem spread, encompassing both evolutionary and transmission drivers. By characterizing these critical transmission pathways and underlying drivers, this review provides an integrated framework to identify critical transmission risks and inform integrated strategies for mitigating antimicrobial resistance across One Health domains.

## 1. Introduction

The discovery and application of antibiotics is undoubtedly a milestone in the field of modern medicine, greatly improving human health and animal welfare. However, antibiotic misuse has accelerated the growing worldwide phenomenon of antimicrobial resistance (AMR), which in turn increased the complexity and failure rates of treatment. AMR has now turned into a major global public health challenge. Global projections forecast that AMR could lead to approximately 1.91 million direct deaths by 2050 [1,2].

The presence of AMR is driven by the carriage of antibiotic resistance genes (ARGs). Antibiotic pressure has dramatically accelerated the spread and evolution of ARGs [3]. Antibiotic residue creates continuous selection pressure, promoting the colonization and dominance of antibiotic-resistant bacteria (ARB) in patients [4,5]. Notably, the routine use of subtherapeutic antibiotic doses in livestock production has also significantly increased the abundance of ARGs in the animals’ gut microbiome [6,7]. These animal-derived ARGs pose a direct threat to human health through the food chain. Residual ARB in meat or dairy can colonize the human digestive tract, and ARGs may transfer to the human gut microbiome with the help of mobile genetic elements (MGEs) [8]. ARGs are increasingly dispersed into the environment through agricultural and household wastewater discharge. Water flow, wind and rain influence the spread and distribution of ARGs in the environment [9]. ARGs in plant-based food mainly originate from contaminated irrigation water or livestock manure used as organic fertilizer. Ultimately, ARGs re-enter humans via food chain, creating a continuous transmission cycle between animals, humans and the environment [10,11]. Importantly, various important multidrug-resistant (MDR) bacteria are prevalent across these four interconnected ecological niches—humans, animals, food, and the environment—as illustrated in Table 1. In this process, ARGs not only spread across bacterial species via conjugative transfer mediated by plasmids, transposons and integrons, but also evolve into novel variants under antibiotic selection pressure [12].

One health emphasizes the inextricable link between humans, animals and the environment to achieve better well-being. The distribution of ARB varies significantly across ecosystems, showing distinct host or environmental preferences, while ARGs can transcend these barriers through MGEs. ARGs have emerged as a novel class of contaminants capable of cross-boundary dissemination because they exploit these interconnected ecological interfaces. However, existing studies have predominantly focused on ARGs surveillance within isolated compartments, largely neglecting their dynamic inter-ecosystem transmission pathways. This study systematically searched medical and environmental databases including PubMed, Web of Science, and CNKI to integratively analyze the occurrence patterns of ARGs and ARB across human, animal, food and environmental domains. To address this gap, this review systematically consolidates ARGs’ occurrence patterns across various domains and analyzes their evolutionary and transmission mechanisms, aiming to provide the theoretical basis for ARG control under the OneHealth framework.

## 2. ARGs/ARB Characteristics Across Various Ecosystems

**Figure 1:**
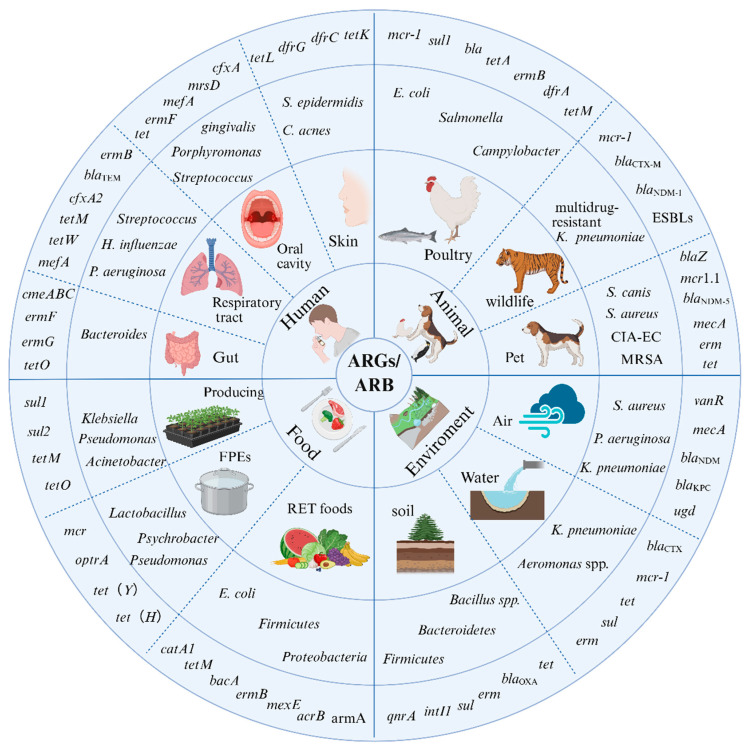
The ARGs/ARB Landscape in Four Key Ecosystems. This multi-concentric circle diagram is divided into four quadrants, representing the interconnected Human, Animal, Food, and Environment ecosystems under the “One Health” framework. Moving from the inner to the outer rings: The second ring details the specific matrices or niches within each ecosystem, including skin/gut/respiratory tract for humans, food-producing animals/wildlife/pets for animals, production/processing/ready-to-eat stages for food, and soil/aquatic/air environments for the environment. The third ring identifies the specific bacteria or ARB prevalent in those niches. The outermost ring lists the corresponding ARGs carried by those bacteria. Created in BioRender. lee, l. (2026) https://BioRender.com/kayztmx (accessed on 8 March 2026).

The widespread use of antibiotics has led to the emergence of ARB in diverse ecological environments, including human, clinical settings, animals, food, water systems and soil (Figure 1). Notably, the dominant antibiotics used in these environments are different. β-lactam and fluoroquinolone antibiotics are commonly used in clinics, while tetracyclines and macrolides are commonly used in livestock farming. This has led to different selection pressures, driving different evolutionary trajectories of ARBs in each ecological environment. Despite these evolutionary differences, ARGs can overcome species barriers through MGEs, enabling cross-ecological dissemination. As a result, ARGs transmission has formed a complex and interconnected network, amplifying the global public health crisis.

### 2.1. ARGs/ARB in the Human

The human hosts a complex ecosystem of distinct microbiomes, spanning the gastrointestinal tract, oral cavity, respiratory tract, skin and so on. Within this system, persistent interactions among microorganisms, the host, and external environmental factors facilitate the emergence and spread of ARGs.

The gut microbial communities harbor approximately 1000 bacterial species and provide 150-fold more than the human genome, often termed the “second human genome” [39]. A large-scale analysis of 5230 global samples indicates that the human gut microbiome can be categorized into a limited set of core “enterosignatures”—stable consortia dominated by specific genera such as *Bacteroides*, *Bifidobacterium*, or *Prevotella* [40]. This finding indicates that beneath the rich diversity of gut microorganisms lies a finite set of stable community structures. However, antibiotic use profoundly disrupts the gut microbiota, leading to reduced diversity, functional alterations, and potential metabolic disturbances [41,42]. For instance, both prolonged antibiotic treatment (e.g., anti-tuberculosis therapy) and short-term combination regimens substantially alter gut microbiota composition [43,44]. A commonly observed change is the increased abundance of *Bacteroides* [43,44]. This finding reveals that specific antibiotics can promote the colonization of certain antibiotic-resistant bacteria by selectively altering the gut microbiota. For instance, five antibiotics, including metronidazole, play a key role in facilitating the intestinal colonization of vancomycin-resistant *enterococci* (VRE). The underlying mechanism involves increasing the availability of multiple nutrients while reducing the production of microbial metabolites, thereby disrupting the colonization resistance against VRE in the gut [35]. These antibiotics compromise the gut’s defensive barrier against VRE colonization by enhancing nutrient accessibility and diminishing microbial metabolic byproducts. This mechanism also sheds light on the successful colonization and transmission of the multi-drug-resistant *Escherichia coli* ST131 clone. As one of the most globally successful extra-intestinal pathogenic clones, ST131 exhibits a high rate of intestinal colonization in healthy community populations, with persistent carriers maintaining long-term colonization, thereby establishing itself as a significant reservoir for household and community transmission [45,46]. The gut microbiota not only serves as a persistent niche for resistant bacteria such as ST131 but also significantly drives the dissemination and evolution of resistance genes through horizontal gene transfer [19,20]. For instance, longitudinal tracking has revealed that ST131 plasmids harboring *bla*_CTX-M-15_ and *qnr* can dynamically transfer between different strains within the patient gut and undergo cross-niche transmission between intestinal and uropathogenic bacteria [47,48]. Similarly, the *Klebsiella pneumoniae* ST258 clone, a globally disseminated carbapenem-resistant lineage, carries the *bla*_KPC-2_ gene. This gene not only persists during intestinal colonization (constituting an important reservoir for nosocomial transmission) but also facilitates infections in the bloodstream, urinary tract, and respiratory system [49,50]. This further confirms the central role of the gut microbiome in the systemic dissemination of drug-resistant bacteria. More notably, there are a large number of horizontal gene transfer events in the human gut microbiota, occurring extensively among both commensal and opportunistic pathogenic bacteria [51,52,53]. In addition, the rate of bacterial horizontal gene transfer is approximately 25 times higher in the human gut microbiota compared to other ecosystems such as soil [54].

The human oral microbiome represents the second largest and most diverse microbiome after the gut [55,56]. Early microbiome studies identified more than 700 bacterial species, more than 100 fungal genera [57], and more than 60,000 species-level phage groups in this niche [58]. Numerous studies have established the oral cavity as a critical ecological niche for the harboring and dissemination of ARGs. This prevalence is evidenced by the consistent detection of ARGs across diverse oral sites—including saliva, gingival crevicular fluid, dental pulp, and mucosal surfaces—in both healthy individuals and those with oral diseases such as dental caries and periodontitis [59,60,61]. Commonly identified ARGs in the oral microbiome include those conferring resistance to macrolides (e.g., *msrD*, *mefA*, *ermF*), β-lactams (e.g., *cfxA*), and tetracyclines (e.g., *tet*) [59,62]. From a microbial vector perspective, resident oral species such as *Streptococcus mitis* and *Porphyromonas gingivalis* have been reported as frequent carriers of these ARGs [59,62]. Importantly, the dynamics of this oral resistome have been characterized: ARGs can be detected as early as infancy, and their diversity and abundance generally follow an accumulative and increasing trajectory with host age [63].

The human respiratory microbiome consists of bacteria, viruses, and fungi that interact with the host via the gut–pulmonary and mouth–pulmonary axes to influence immune and inflammatory responses in the lungs [64]. Prolonged treatment with antibiotics leads to a decrease in the diversity of the respiratory microbiome and promotes the enrichment of drug-resistant bacteria. Commonly resistant bacteria include *Pseudomonas aeruginosa* (*P. aeruginosa*), *Haemophilus influenzae* (*H. influenzae*), and *Streptococcus*, which show varying degrees of resistance to β-lactam, macrolide and fluoroquinolone antibiotics [65]. It has also been found that there is a “core resistome” of respiratory resistomes, including β-lactamase genes (e.g., *bla*_TEM_ and *cfxA2*), macrolide-resistant genes (e.g., *ermB* and *msrD*), and tetracycline-resistant genes (e.g., *tetM* and *tetW*), which are prevalent in healthy individuals and patients with chronic respiratory diseases [66]. In addition, long-term use of macrolides (e.g., azithromycin) leads to an enrichment of resistance genes in the respiratory microbiome, especially an increase in genes such as *ermB* and *mefA*, further exacerbating the problem of antibiotic resistance [67,68]. *Moraxella catarrhalis*, once considered a harmless commensal organism of the upper respiratory tract, is now recognized as a significant pathogen, particularly in patients with chronic obstructive pulmonary disease (COPD) [69]. Critically, nearly all clinical isolates of *M. catarrhalis* harbor the *BRO* β-lactamase genes *(BRO-1* or *BRO-2*), which confer penicillin resistance and contribute to the respiratory resistome [70].

Not only is human skin an important barrier against external aggressions, but it is also home to a variety of commensal microorganisms, including viruses, fungi and bacteria. Research has found that, in the absence of antibiotic intervention, the structure of bacterial, fungal and viral skin communities remains remarkably stable over observation periods of months or even years, despite continuous exposure to a complex external environment [71]. However, mainstream therapeutic regimens for common dermatopathologic conditions, such as acne and rosacea, as well as for cutaneous soft-tissue infections, are still highly dependent on the clinical use of broad-spectrum antibiotics [72,73]. This dependence inevitably disrupts the equilibrium of the skin microbiota, thereby promoting the emergence and proliferation of antibiotic-resistant strains. Among these, methicillin-resistant *Staphylococcus aureus* (MRSA) has become a major threat in skin colonization and infection. As the most common pathogen responsible for skin and soft tissue infections (SSTIs), MRSA is widely colonized in community and hospital settings on the skin and its associated structures, including the anterior nares and perianal skin [31,32]. Notably, MRSA colonization at these skin sites establishes a reservoir; upon disruption of the skin barrier, microbiome imbalance, or immunosuppression, it can rapidly transition from a commensal state to invasive infection, thereby rendering it one of the most prevalent drug-resistant pathogens in skin and soft tissue infections globally [74,75,76].

A controlled study on healthy volunteers revealed that treatment with antibiotics commonly used for skin infections—such as doxycycline, cephalexin, and trimethoprim/sulfamethoxazole—significantly disrupted the skin microbiome and increased the relative abundance of resistant staphylococci, such as *Staphylococcus epidermidis* (*S. epidermidis*). These strains carried ARGs such as *tetK*, *tetL*, *dfrC*, and *dfrG*, and their dominance persisted even after the antibiotics were withdrawn [77]. Furthermore, during the use of antibiotics for the treatment of skin-associated infections, a variety of skin-colonizing flora have shown significant antibiotic resistance, including *Cutibacterium acnes* (*C. acnes*). Notably, *C. acnes* has been reported to have more than 50% resistance to erythromycin, clindamycin, doxycycline, and tetracycline [78,79].

Within the human urinary microbiome, *Escherichia coli* (*E. coli*) and *Proteus mirabilis* (*P. mirabilis*) are the two most common pathogens associated with urinary tract infections (UTIs) and are facing increasingly severe antimicrobial resistance. *E. coli*, as the primary pathogen responsible for both community- and hospital-acquired UTIs, has exhibited a significant increase in resistance to fluoroquinolones, trimethoprim-sulfamethoxazole, and extended-spectrum cephalosporins (ESCs) [80]. *P. mirabilis*, on the other hand, is an important pathogen in complicated UTIs, particularly catheter-associated UTIs, and similarly confronts substantial resistance challenges. Resistance to ESCs in both species is primarily mediated by plasmid-encoded β-lactamase genes. The major resistance mechanism involves extended-spectrum β-lactamases (ESBLs) from the TEM, SHV, and CTX-M families, with plasmid-mediated AmpC β-lactamases (p-AmpC) playing a secondary role [13,14,15]. The horizontal transfer of these resistance genes via plasmids not only facilitates the widespread dissemination of multidrug-resistant phenotypes but also enables the long-term colonization of resistant strains within the urinary tract, thereby establishing a critical reservoir for refractory infections and posing a severe threat to clinical anti-infective therapy [81].

In addition, studies have shown that the problem of bacterial resistance in eye infections is increasing. Common ocular pathogens such as *Pseudomonas aeruginosa* and *Staphylococcus aureus* have demonstrated high resistance to fluoroquinolones and methicillin. ARGs are transmitted between bacteria by horizontal gene transfer, making the treatment of ocular infections increasingly difficult [82]. Overall, the selective pressure of antibiotics drives the emergence and dissemination of ARB and ARGs across diverse human body sites. These resistant entities exist on a spectrum from silent colonization to active infection, fundamentally complicating treatment outcomes.

### 2.2. ARGs/ARB in the Animal

The distribution of ARB and their corresponding resistance genes in animals exhibits notable host-specific and ecology-dependent characteristics. Recent studies on companion animal skin microbiota have revealed distinct microbial profiles between healthy and diseased states. The skin microbiome of healthy dogs is predominantly composed of *Streptococcus* spp., whereas inflammatory skin lesions are primarily colonized by *Staphylococcus* spp. [83]. Antimicrobial resistance is frequently detected in staphylococci isolated from skin disorders of companion animals. Research on skin and soft tissue infections (SSTIs) has shown that *Staphylococcus aureus* (*S. aureus*) isolates from infected animals exhibit a high level of resistance, with over half (56.4%) identified as methicillin-resistant *S. aureus* (MRSA) [84]. Similarly, *S. pseudintermedius* associated with SSTIs in companion animals is predominantly methicillin-resistant (MRSP) [85]. These resistant strains have been reported to carry resistance genes such as *blaZ*, *mecA*, and *erm* [84,85]. Apart from pathogens associated with skin infections, antimicrobial resistance within the gut microbiota of companion animals also warrants significant attention. A recent survey involving 11 veterinary hospitals in Shanghai reported the isolation of 244 critically important antibiotic-resistant *Escherichia coli* (CIA-EC) strains from 730 anal swab samples. These isolates were found to harbor a variety of ARGs, including *bla*_NDM-5_, *mcr*1.1, the fluoroquinolone resistance gene *qepA*, and *tet*(X4), among others [86]. Moreover, the oral microbiome of healthy dogs has been shown to host a broad spectrum of resistance genes, including those conferring resistance to macrolides, tetracyclines, and lincosamides [87]. This suggests that the oral cavity may serve as an important micro-ecological niche for the storage and dissemination of ARGs. Furthermore, the antimicrobial resistance of *Streptococcus canis* (*S. canis*), one of the most frequently isolated streptococcal pathogens from various infections in companion animals, cannot be overlooked. *S. canis* isolates from dogs and cats commonly exhibit resistance to antimicrobial agents commonly used in veterinary and human clinical practice. Studies have revealed that approximately 67.7% of the isolates were resistant to at least one antimicrobial agent, among which 56.9% carried multiple transferable resistance genes. These primarily include the *tet* gene family, conferring resistance to tetracyclines, and the *erm* genes, responsible for resistance to macrolides–lincosamides–streptogramin B (MLSB) [88]. However, the threat posed by companion animals as reservoirs of resistance genes extends far beyond this. Although carbapenems are not approved for use in veterinary medicine, carbapenem-resistant Enterobacteriaceae (CRE) carrying resistance genes such as *bla*_KPC_, *bla*_NDM_, and *bla*_OXA-48-like_ have been increasingly reported in companion animals worldwide [18,19,20,21]. This paradoxical finding strongly suggests that companion animals have emerged as potential reservoirs and transmission vectors for these clinically significant antibiotic resistances.

Compared to companion animals, the distribution and transmission of ARGs in wildlife also warrant significant attention. Studies have shown that migratory birds carry ARGs with high diversity and abundance. One investigation detected 130 distinct ARGs, categorized into 202 resistance types, conferring resistance primarily to tetracyclines, aminoglycosides, β-lactams, and sulfonamides. Notably, the *mcr-1* gene was detected in 50% of the wild bird samples, indicating its widespread prevalence [89]. Beyond avian species, high-risk resistant bacteria have also been reported in various wild mammals in Africa, such as primates and carnivores. Yang et al. [90] identified multidrug-resistant *Klebsiella pneumoniae* strains carrying *bla*_CTX-M-15_ and *bla*_NDM-1_ genes in these populations, whose genomes were highly homologous to human clinical isolates. This suggests that contamination from medical waste or ecotourism may facilitate the transmission of resistance genes from humans to wildlife. Aquatic environments also serve as important mediators for the spread of resistance in wildlife. Thibodeau et al. [91] highlighted that wild freshwater fish and aquatic mammals can carry ARGs such as ESBLs and *mcr-1* through bioaccumulation, acting as effective bioindicators of aquatic resistance pollution. More alarmingly, even in the highly geographically isolated Antarctic ecosystem, Gutiérrez et al. [92] detected β-lactamase genes and fluoroquinolone resistance mutations in wildlife such as penguins and seals. The similarity between the identified ARGs and those in human clinical strains suggests that cross-ecosystem gene flow may have transcended geographical barriers [93].

Unlike wildlife and companion animals, the distribution of ARB and ARGs in farmed animals within aquaculture and livestock systems is directly governed by human management practices. Their spatial and temporal patterns are closely linked to the effectiveness of antibiotic use control. Global surveillance data reveal significant geographic heterogeneity and drug-selective pressure in the prevalence of resistant bacteria among livestock. Zhao et al., through the development of antibiotic priority maps, reported that *E. coli* from poultry and swine in Africa and Asia exhibited resistance rates of 30–60% to fluoroquinolones and third-generation cephalosporins [94]. Further studies indicate that in Europe, *Salmonella* and *Campylobacter* from poultry and swine demonstrate resistance rates exceeding 50% to tetracyclines and sulfonamides [95]. These values showed a significant positive correlation with regional antibiotic misuse indices. Notably, the enrichment of ARGs in farming environments exhibits clear spatiotemporal persistence. This persistence is not only reflected in the environmental medium but also directly within the animal host. Peng et al. detected multidrug-resistant *E. coli* carrying *bla*_CTX-M_ and *mcr-1* genes in animal feces and surrounding soils, indicating that ARGs can persist and disseminate widely within the environment [96]. Direct surveillance of farm animals has further confirmed the pervasiveness of this issue: multiple studies have consistently reported the widespread occurrence of CTX-M-positive and mcr-positive *Escherichia coli* in poultry and other livestock, indicating that these resistance genes have become established as stable reservoirs within farm animal populations [97,98,99]. Complementing this, Juricova et al. revealed a dose-dependent accumulation of *tetA* and *ermB* genes in chicken gut commensals, suggesting that feed additives may facilitate the intergenerational accumulation of ARGs through vertical transmission pathways [100].

In aquaculture systems, similar trends of ARG enrichment driven by antimicrobial usage have been documented. Macro-epidemiological data reveal that the multiple antibiotic resistance (MAR) index of bacteria in aquaculture systems shows a significant correlation with that of clinical human isolates, suggesting that antimicrobial use in human medicine, livestock, and aquaculture collectively contributes to a shared resistome [101]. At the molecular level, this pattern is further substantiated: metagenomic analyses have uncovered a diverse array of ARGs in aquaculture environments—including genes conferring resistance to sulfonamides, tetracyclines, aminoglycosides, and fluoroquinolones—whose abundance is directly linked to antimicrobial selection pressure [102]. More focused investigations in shrimp farming environments have demonstrated a high prevalence of ceftriaxone-resistant bacteria in both water and shrimp samples. Molecular characterization of isolated strains confirmed the widespread presence of β-lactam resistance genes, such as *bla*_CTX-M_, *bla*_SHV_, *bla*_OXA_, and *bla*_TEM_, which mediate resistance to third-generation cephalosporins [103]. In summary, evidence spanning macro-epidemiological associations to micro-level genetic mechanisms consistently indicates that antimicrobial-driven selection enriches ARGs in aquaculture.

These findings collectively demonstrate that antimicrobial resistance in animals is mainly shaped by human activities. In companion animals and farmed animals, antimicrobial resistance manifests as direct selective pressure from antibiotic use, while in wild animals, it reflects ecological diffusion caused by human pollution, forming an interconnected network of resistance.

### 2.3. ARGs/ARB in the Food

Food safety remains a pivotal concern in global public health. As research on the pathogenesis of foodborne illnesses advances, the role of food as a potential vehicle for the dissemination of ARGs has garnered increasing attention. Accumulating evidence indicates that various food types can serve as significant reservoirs and transmission routes for ARGs to humans [104]. Foods of animal origin, dairy products, and eggs often harbor antibiotic residues originating from therapeutic or prophylactic use in livestock production [105,106]. Similarly, plant-derived foods are not exempt from ARG transmission risks, particularly when cultivated using irrigation water contaminated with antibiotics or when grown in soils amended with manure or fertilizers containing antibiotic residues [107,108]. Notably, ARGs can disseminate throughout the entire food supply chain, spanning agricultural production, animal husbandry, food processing, distribution, and final consumption [109].

Within agricultural systems, soil acts as a critical reservoir and dissemination hub for ARGs, whose accumulation and spread can subsequently influence crops [110,111,112]. One long-term study demonstrated that 40 years of manure application led to a linear increase in the abundance of sulfonamide (*sul1*, *sul2*) and tetracycline (*tetM*, *tetO*) resistance genes in soil, with ARG enrichment in the crop rhizosphere reaching 3–8 times that of unaffected soils [113]. Furthermore, studies have identified potential pathogenic bacteria—such as *Pseudomonas*, *Klebsiella*, and *Acinetobacter*—carrying multidrug resistance genes and β-lactam ARGs in the phyllosphere of fresh vegetables [113,114]. Notably, some culturable strains from these environments exhibited resistance to clinically critical antibiotics, including colistin and meropenem [108]. It is also noteworthy that although organic farming systems support agricultural sustainability, long-term application of organic fertilizers has been shown to increase the number of diverse ARG-MRG-carrying contig types in the soil microbiome by threefold compared to conventional farming, based on an analysis of 511 global agricultural soil metagenomes [115]. In addition, the diversity and abundance of ARGs in the phyllosphere of organically grown lettuce were significantly elevated [107,116]. Moreover, the persistence and dynamic changes in ARGs such as *tetX*, *tetO*, *tetG*, and *sul2* during composting suggest that these genes may re-enter the food chain via the organic fertilizer–crop transmission route [117], underscoring the need to monitor and manage the cycling of resistance genes in organic agricultural practices.

Food processing environments (FPEs) represent critical reservoirs and dissemination hotspots for ARB and ARGs [118]. Studies have shown that in pork processing plants, both the abundance and diversity of ARGs increase during operational hours, with genes conferring resistance to β-lactams, tetracyclines, aminoglycosides, and fluoroquinolones being predominant [119]. Similarly, in beef processing chains, substantial contamination with ARGs has been detected on carcasses and processing surfaces, predominantly involving aminoglycoside, β-lactam, and tetracycline resistance genes (*tet(H)*, *tet(Y)*) [120]. Many of these ARGs are located on mobile genetic elements, indicating a high potential for horizontal gene transfer [120]. A study conducted in a Chinese swine slaughterhouse revealed the persistent presence of multidrug resistance genes and β-lactamase genes across the production chain—from fecal matter and processing environments to the final pork products [121]. Clinically critical resistance determinants such as *mcr* and *optrA* were also identified [121]. The continuous detection of ARGs, virulence factors, and mobile genetic elements throughout the processing stages suggests an ongoing flow of resistance genes along the production chain. Furthermore, microbial community structures in different FPEs exhibit sector-specific characteristics: *Pseudomonas* and *Psychrobacter* dominate in slaughterhouses and meat processing plants, while *Lactobacillus* is more prevalent in dairy facilities [122]. Correspondingly, the profiles of ARGs are environment-dependent. Aminoglycoside and tetracycline resistance genes are more common in slaughterhouses, whereas quaternary ammonium compound (QAC) resistance genes are enriched on contact surfaces in dairy and meat processing plants—implying that routine disinfection practices may also exert selective pressure favoring tolerant microbial populations [122]. Despite stringent hygiene controls and processing protocols in food production environments, ARB and ARGs may still enter final products through direct or indirect contamination. A study of ready-to-eat foods (RET foods) in southern China detected a variety of ARB and ARGs. The identified ARB primarily included *Proteobacteria* and *Firmicutes* [123]. The study also revealed multiple ARGs, namely *catA1*, *acrB*, *APH3IIIA*, *bacA*, and *tetM*, conferring resistance to chloramphenicol, multiple drugs, aminoglycosides, bacitracin, and tetracyclines, respectively [123].

Thermal processing during cooking can effectively eliminate viable ARB; insufficient heating or post-processing contamination may allow ARB and associated genetic determinants to persist [124]. Several studies have successfully extracted ARGs, including *armA* and *sul1*, as well as resistant *E. coli*, from the DNA of cooked food products [125]; however, the impact of thermal treatment on the integrity and functional potential of ARGs remains inadequately investigated. It is commonly recognized that refrigerated storage better preserves food quality compared to ambient conditions, as low temperatures inhibit spoilage and slow the growth and reproduction of antibiotic-resistant microorganisms [126]. Nevertheless, it is important to emphasize that while refrigeration suppresses microbial activity, it does not fully eliminate the presence or potential dissemination of ARGs [127]. Multiple ARGs have been detected in foods stored in domestic refrigerators. One study identified up to 134 distinct ARGs and MGEs in vegetables, fish, and pork [128]. These genes were subjected to risk assessment based on criteria such as human accessibility, genetic mobility, host pathogenicity, and clinical relevance [129]. The results indicated that high-risk ARGs accounted for 10.06% of the total, including genes such as *tetM*, *aadA5*, and *mexE* [128]. Of particular concern, fish samples exhibited a rapid increase in the abundance of ARGs and pathogenic bacteria after only three days of storage, indicating that everyday food storage environments could act as sites for the “secondary enrichment” of ARGs [128].

### 2.4. ARGs/ARB in the Environment

ARB are ubiquitously present across diverse environmental compartments. The occurrence and distribution characteristics of ARGs in ambient air vary considerably across different environments. Healthcare settings represent one of the most prominent hotspots for airborne ARG contamination. The average concentration of ARGs in hospital air has been reported to be substantially higher than in drinking water, wastewater, and marine environments [130]. A variety of clinically relevant ARGs, such as glycopeptide resistance genes *vanR* and *ugd* and carbapenem resistance genes *bla*_NDM_ and *bla*_KPC_, have been detected in indoor hospital air [131]. Moreover, beyond healthcare facilities, wastewater treatment systems also constitute significant reservoirs of ARGs in aerosols. In the aeration tanks of urban wastewater treatment plants (WWTPs), 44 ARG subtypes were commonly detected in both aerosol and activated sludge samples [132]. The detection frequency of β-lactam and tetracycline resistance genes in the aerosol phase was 12.7–18.4% higher than in liquid samples [132]. More importantly, studies indicate that WWTPs are major sources of ARGs and ARB such as *S. aureus*, *P. aeruginosa*, and *K. pneumoniae* in the surrounding air. Processes such as aeration can lead to the aerosolization and release of the resistome into the atmosphere, significantly impacting local air quality [133,134]. For instance, previous research has shown that 11–13% of bacteria and 30–57% of ARGs in PM_2.5_ around urban and WWTP areas can be traced back to wastewater treatment facilities [135]. The abundance of ARGs and clinically relevant genes in PM_2.5_ increases along a gradient from coastal areas to urban sites and WWTPs. Additionally, studies on food wastewater treatment plants (FWTPs) reveal that although the total abundance of ARGs in PM_2.5_ does not differ significantly from that in municipal WWTPs, PM_2.5_ from FWTPs is significantly enriched with multidrug resistance genes [136]. The resulting exposure risk to resistant pathogens can be 5–11 times higher than that from urban ambient PM_2.5_ and even 41.53% higher than in hospital settings [136]. Similarly, various ARGs *mecA* originating from agricultural activities have been detected in the air surrounding livestock farms, with impacts extending up to 10 km, forming regional-scale pollution zones [137]. Although current estimated doses of ARG exposure via ingestion—such as through drinking water and food—still exceed those via inhalation, the risk posed by aerosol-mediated respiratory exposure can no longer be overlooked.

Healthcare settings, livestock farms, and wastewater treatment plants serve not only as emission sources of airborne ARGs, but also as major contributors of ARGs in aquatic systems. Wastewater discharges from these sites represent a primary cause of ARG contamination in surface water, groundwater, and even drinking water sources. Hospital wastewater has been identified as a key node for the spread of antibiotic resistance in aquatic environments [138]. A systematic review showed that the absolute abundance of ARGs in raw hospital wastewater was 1–2 orders of magnitude higher than in community wastewater and carried more clinically relevant resistance genotypes, *bla*_CTX-M_, *mcr-1* and so on [139,140]. Metagenomic sequencing further revealed the presence of RND-type efflux pump gene cluster *tmexCD-toprJ*, in hospital sewage systems. This cluster predominantly originates from *Aeromonas* spp., commonly found in aquatic environments, and can be transferred to clinically pathogenic CRKP, causing tigecycline resistance [141,142]. WWTPs, owing to their pivotal role in urban and industrial water cycles, are recognized as critical hotspots for the dissemination of ARGs into aquatic environments [143]. The influent entering WWTPs receives wastewater from diverse urban, industrial, and domestic sources, where various ARGs—including those conferring resistance to macrolides (e.g., *ermB*, *ermF*), tetracyclines (e.g., *tetW*, *tetA*, *tetC*), sulfonamides (e.g., *sul1*, *sul2*), and β-lactams—can be detected [144]. Furthermore, studies analyzing untreated and treated effluents from WWTPs as well as upstream and downstream river water have shown that although the concentrations of ARB and ARGs are substantially reduced during wastewater treatment, significant quantities are still released into the environment [145]. In addition, various common antibiotics—such as sulfonamides, tetracyclines, and chloramphenicol—along with diverse ARGs have been detected in aquaculture systems and their effluent discharges using advanced analytical techniques [146,147]. Ultimately, antibiotic residues, resistant bacteria, and ARGs originating from hospitals, urban centers, and aquaculture operations are discharged into rivers, lakes, coastal waters, and groundwater systems, leading to widespread contamination [148]. Studies have identified 203 unique ARGs and 10 types of MGEs in landfill leachate and adjacent groundwater, with approximately 96% of the ARGs traced back to the nearby landfill leachate [149]. Moreover, quantitative analysis of multiple ARGs in drinking water samples from several Chinese cities revealed that sulfonamide resistance genes were the most frequently detected type, while the *bla*_TEM_ gene, ranking among the top five most abundant ARGs, requires heightened attention due to its clinical relevance [150]. Freshwater lakes in China have also become significant reservoirs of antibiotics and ARGs. National monitoring data indicate the widespread presence of dozens of antibiotics, including sulfonamides and tetracyclines, as well as *sul*-type ARGs in lacustrine environments across the country [151,152].

Beyond aquatic environments, soil represents another major environmental medium that receives and enriches ARGs. ARGs originating from medical, aquaculture wastewater, and domestic sewage are introduced into soil through irrigation, sedimentation, and other pathways, where they persist long-term, proliferate, and constitute a unique soil resistome. Agricultural activities are a significant cause of soil ARG contamination, with two primary input pathways currently identified: (1) Application of organic fertilizers: Manure-derived microorganisms (particularly ARG-host bacteria such as *Bacillus* spp.) alter the soil microbial community structure through colonization, leading to increased diversity and abundance of ARGs. (2) Reclaimed water irrigation: ARGs not fully removed enter the soil system with recycled water [153]. Keenum et al. found that the combined effect of cattle manure compost treatment and antibiotics had a dual impact on ARG abundance: although thermophilic composting could degrade some antibiotics and reduce *tetM* and *ermB* via microbial metabolism, other genes such as *sul1*, *intI1*, β-lactam ARGs, and plasmid-associated genes increased [154]. Peng et al. analyzed soils with long-term application of different manures and found that 30 years of continuous swine manure application significantly increased the abundance of ARGs (e.g., *tetQ* and *sul2*) in soil, along with a marked enrichment of key ARB such as *Firmicutes* and *Bacteroidetes*. This enrichment was substantially higher than that observed in poultry manure and chemical fertilizer treatments [155]. This was closely related to the higher residual antibiotic concentrations in swine manure and alterations in the microbial community structure. Zhang et al. further confirmed through a 40-year long-term positioning experiment that manure application led to a linear or exponential increase in soil ARGs (e.g., *tetA* and *ermF*) [113]. Even after manure application ceased, these ARGs could persist in the soil for extended periods. Furthermore, Shekhawat et al. discovered that in irrigated soils near a specific institutional wastewater treatment plant, the abundances of aminoglycoside, β-lactam, and sulfonamide ARGs were significantly higher than in control soils, potentially related to the wastewater source (containing hospital effluent) and the selective survival of resistant bacteria due to UV disinfection processes [156]. Irrigation with specific types of wastewater may directly alter the resistance profile of the soil microbiome by introducing resistant bacteria or free ARGs. Similarly, landfill leachate serves as a significant pollution pathway, introducing ARB and ARGs into surrounding soils. Studies have confirmed that leachate often contains elevated concentrations of antibiotics, such as fluoroquinolones and β-lactams, along with their corresponding ARGs (e.g., *qnrA* and *bla*_OXA_) [157]. In many landfills, the relative abundance of ARGs, such as *qnr*, *erm*, and *sul*, can reach or exceed 10^−4^ copies per 16S rRNA, indicating significant contamination, a spread primarily mediated by MGEs [158]. Consequently, landfills are regarded as critical reservoirs and sources of ARGs and ARB, posing persistent ecological and public health risks to adjacent soils and groundwater [159,160].

While aquatic, soil, and atmospheric environments are recognized as major sinks for antibiotics and ARGs, a growing body of evidence underscores that solid surfaces in various human-made settings constitute important reservoirs and transfer hotspots for ARGs. In healthcare facilities, environmental surface swab data indicate that high-touch surfaces—such as nursing stations, patient room sinks, and department door handles—are frequently contaminated with resistance genes associated with β-lactams, fosfomycin, and cephalosporins. Notably, the abundance of *bla*_CTX-M_ detected on intensive care unit (ICU) surfaces was found to be 3.2 times higher than that in general wards [161]. Moreover, multiple surfaces in nursing home environments have been shown to harbor multidrug-resistant organisms (MDROs), highlighting the widespread nature of ARG contamination in healthcare-associated settings [162]. In community and public environments, routine and intensive disinfection of surfaces may exert persistent selective pressure on local microbial communities. This not only enhances their tolerance to disinfectants but may also indirectly co-select for resistance to certain antibiotics [163]. For example, during the COVID-19 pandemic, frequent disinfection of elevator buttons across different venues was associated with the enrichment of resistant bacteria and a corresponding increase in the abundance of relevant ARGs [164].

## 3. Dissemination and Evolution of ARGs Across Different Domains

The threat of ARGs lies not only in their existence, but equally in their remarkable capacity for mobility and adaptation. Powered by strong evolutionary pressures, ARGs spread and evolve within ecological and human domains, aided by diverse and highly efficient genetic transfer mechanisms. Unraveling this interconnected chain, from evolutionary drivers, to dissemination mechanisms, and finally to transmission routes, is essential for anticipating and ultimately containing the global expansion of antimicrobial resistance.

### 3.1. Evolutionary and Transmission Drivers of ARG

The evolutionary origins of ARGs are deeply rooted in ecological and evolutionary history. Molecular evidence indicates that many ARGs originated from adaptive strategies developed by microorganisms in response to competitive pressures within natural environments [165]. Long before the industrial-scale production of antibiotics by humans, bacteria had already evolved sophisticated resistance mechanisms through interspecies competition and co-evolution. Paleogenomic analyses of Siberian permafrost by D’Costa et al. revealed that microbial communities harboring genes encoding β-lactamase and vancomycin resistance existed as early as 30,000 years ago [166]. This finding was further supported by functional metagenomic studies, such as that of Perron et al., which reconstructed ancient gene clusters with tetracycline-modifying enzyme activity from 5000-year-old Arctic soil microbiomes [167]. Moreover, isolates obtained from 3.5-million-year-old permafrost have yielded staphylococcal strains carrying multiple resistance determinants [168]. Many bacteria retain chromosomally encoded intrinsic resistance genes—for instance, *Aeromonas* species harbor the *bla*_CphA_ gene and *Shewanella* species carry *bla*_OXA-like_ genes—which can be regarded as molecular imprints of ancient adaptive strategies. However, these environmental organisms are no longer merely passive carriers of intrinsic resistance determinants. Under modern selective pressures, they are now engaging in frequent genetic exchanges with clinically derived bacteria and plasmids, acquiring and stably integrating high-risk resistance genes such as *bla*_NDM_ or *mcr* [169]. Collectively, these ancient records suggest that microbial competition for survival served as a key driver in the early evolution of resistance traits, with natural selection pressures playing a central role in shaping these adaptive pathways (Table 2) [170,171].

**Figure 2:**
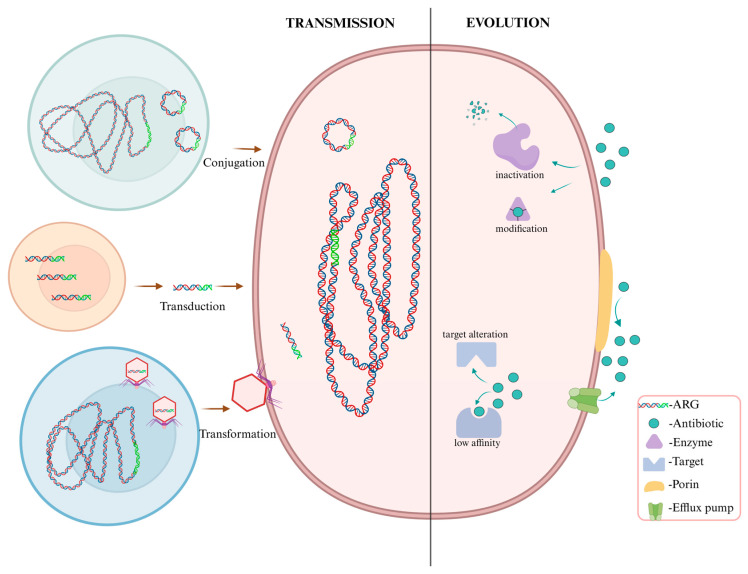
Mechanisms of the Transmission and Evolution of ARGs and ARB. Created in BioRender. lee, l. (2026) https://BioRender.com/v5uqimn (accessed on 8 March 2026).

In the modern era, human activities—such as the large-scale application of antibiotics in agriculture, animal husbandry, and clinical practice—have introduced novel and sustained selective pressures that profoundly accelerate the evolution and dissemination of ARGs [172]. Under such high and persistent antibiotic exposure, the development of resistance represents a central adaptive strategy for bacterial survival. The evolutionary mechanisms underpinning this adaptation include (Figure 2): (1) enzymatic inactivation or modification of antibiotics; (2) structural alteration of drug targets or reduced target affinity; (3) modulation of membrane permeability through efflux pump activation or porin downregulation [173]; (4) chromosomal mutations, including adaptive changes in core metabolic genes [174]; and (5) horizontal gene transfer (HGT). Among these, HGT has been identified as a major driver in the rapid dissemination of ARGs across bacterial species and environments [175]. These mechanisms frequently operate individually or in concert to shape the evolutionary trajectory of antibiotic resistance in diverse ecological and clinical settings.

In contemporary environments, the dissemination and evolution of ARGs are co-regulated by multiple environmental drivers. Soil acts as a major reservoir of ARGs, where mineral constituents (e.g., quartz, kaolinite, montmorillonite) and organic matter (e.g., humic acid, biochar, soot) significantly influence ARG retention and transformation through physical adsorption or chemical interactions [176]. Notably, combined effects of minerals and organic matter exhibit marked differences: biochar can enhance bacterial co-resistance by inducing intracellular reactive oxygen species (ROS) bursts, whereas kaolinite and montmorillonite may suppress horizontal gene transfer (HGT) of ARGs via nucleic acid adsorption [177]. Moreover, shifts in soil physicochemical properties profoundly shape ARG dynamics: the high specific surface area of montmorillonite under acidic conditions can lead to nutrient depletion and enhance ARG adsorption, thereby restricting their spread [178]. In contrast, microplastic pollution has been shown to increase ARG abundance by altering soil microbial community structure, particularly interactions between rhizospheric and non-rhizospheric microbes, with ARGs linked to MGEs being especially enriched under long-term mulch coverage [179,180].

Beyond terrestrial settings, ARG dissemination in aquatic ecosystems is similarly driven by multiple factors. The distribution and transfer of ARGs in water are jointly influenced by variables such as dissolved oxygen, nutrient concentrations, nanomaterials, and heavy metals [9]. Heavy metals (e.g., Cu, Zn) may promote the selection of multi-resistant strains through co-selective pressure, while oxidants such as hypochlorite can induce bacterial SOS responses, thereby facilitating plasmid-mediated transfer [181,182].

Concurrently, global climate change is exacerbating the cross-media migration of ARGs. Warming temperatures can indirectly accelerate bacterial metabolic rates, promoting ARG emergence and spread [183]. Extreme weather events (e.g., floods, heavy rainfall) further promote the environmental mobility of ARGs. Precipitation acts as a vehicle for ARGs, with their atmospheric deposition to soil being significantly affected by wind speed and particulate matter (PM) concentration [184].

Biological vectors also play a crucial bridging role in ARG transmission. Insects such as houseflies and cockroaches can carry resistant bacteria on their surfaces or in their gut, facilitating the cross-boundary transfer of ARGs between human-dominated and natural environments [185]. The high abundance of MGEs in their intestines may further enhance the efficiency of ARG horizontal transfer. Additionally, the long-distance migratory behavior of wildlife offers a unique route for resistance gene dispersal; for instance, wild birds frequently carry *bla*_TEM_ genes, highlighting their potential role as ARG vectors.

### 3.2. Mechanisms of ARG Dissemination

The dissemination of ARB and ARGs occurs through two primary mechanisms: vertical gene transfer (VGT) and horizontal gene transfer (HGT). VGT refers to the stable inheritance of ARGs—whether plasmid-borne or chromosomally encoded—from mother to daughter cells via binary fission, leading to the clonal expansion of specific resistant lineages [186,187].

In contrast, HGT serves as the principal driver for the rapid dissemination of ARGs across different strains, species, and even genera, enabling recipient bacteria to acquire resistance genes and express corresponding resistance phenotypes [5,188]. HGT operates through mechanisms such as transformation (uptake of free environmental DNA), transduction (phage-mediated gene shuttling), and conjugation (cell-to-cell transfer via plasmids or conjugative elements) (Figure 2) [189].

VGT and HGT do not operate in isolation; rather, they exhibit dynamic interplay and mutual reinforcement. VGT maintains the baseline frequency of ARGs within bacterial populations, whereas HGT enables discontinuous, cross-clonal transmission of resistance determinants [187]. Notably, due to the substantial metabolic cost associated with conjugation, most clinical plasmids maintain relatively low conjugation frequencies, suggesting that natural selection favors optimized VGT to ensure plasmid stability [187]. Under antibiotic selective pressure, these two mechanisms can act synergistically: VGT rapidly expands pre-existing resistant clones, while HGT disseminates resistance genes into novel genetic backgrounds [190].

Among these, plasmids serve as principal vectors in the spread of antibiotic resistance. They not only act as key vehicles for HGT between bacteria but also drive the exchange of ARGs across different ecological niches and habitats [191,192]. As core MGEs, plasmids can spread efficiently via conjugation within bacterial communities. Analysis of 8229 plasmids carrying ARGs revealed highly dynamic capture and recombination of resistance determinants, with approximately 87% showing potential for inter-plasmid transfer [193]. Moreover, over 88% of inferred gene transfer events occurred between compatible plasmids co-residing within the same bacterial host, highlighting a co-evolutionary mechanism within multi-plasmid systems [193]. The propagation of ARGs is further amplified through the interplay between plasmids and other MGEs such as insertion sequences (IS). For instance, 77.3% of plasmids carrying ARGs were found to co-localize with IS elements, and these mobile elements mediated 63.2% of plasmid-to-chromosome ARG transfer events [12]. Thus, beyond directly facilitating ARG dissemination through multi-plasmid cooperation, plasmids also serve as platforms for IS elements and transposons to mobilize ARGs, thereby promoting the spread of resistance across diverse microorganisms and ecosystems. Plasmid-mediated transmission of antibiotic resistance represents a growing global public health challenge. Plasmids drive the spread of ARGs through the establishment of multi-tiered dissemination networks: facilitating interspecies HGT within microbial communities, traversing species barriers to infiltrate distinct bacterial populations, and—via food chain-mediated cross-exposure—bridging ecological niches across animal hosts, the human gut microbiota, agricultural systems, and natural environments. This culminates in a cross-niche transmission system capable of widespread ecological penetration [194,195]. The discovery of the plasmid-borne colistin resistance gene *mcr-1* in 2015 demonstrated that animal-associated plasmids can transfer resistance to human pathogens via HGT, underscoring both the scientific significance and public health impact of such transmission routes [196]. The evolutionary trajectory of *Klebsiella pneumoniae* further illustrates the clinical relevance of plasmid-acquired evolution: classical strains integrating a *bla*_KPC_-carrying resistance plasmid evolve into carbapenem-resistant *K. pneumoniae* (CRKP), whereas acquisition of a pLVPK-like virulence plasmid drives transition toward a hypervirulent phenotype (hvKP) [197]. As MGEs, plasmids not only enable cross-species transfer of resistance genes via conjugation but also function as key evolutionary drivers that promote both the dissemination and long-term persistence of ARGs within bacterial populations [191,198].

Phages represent one of the most abundant biological entities in ecosystems, primarily mediating the dissemination of genetic materials such as ARGs through transduction in HGT. While current research predominantly focuses on transduction pathways, limited evidence suggests that certain phage families may also participate in HGT via mechanisms such as conjugation [199] and transformation [200]. Specifically, phages facilitate the transfer of ARGs into bacterial hosts through generalized transduction—which packages arbitrary genomic fragments from donor bacteria—and specialized transduction, which transfers specific donor genes, by encapsulating ARGs into phage or phage-like particles [201]. Further studies indicate that prophages function as dynamic genetic regulatory units capable of responding to environmental signals and actively influencing the dissemination trajectories of ARGs. For instance, Enterobacteriaceae phages can integrate prophage-encoded ARGs (e.g., *bla*_TEM_ and *qnrS*) into the host genome via lysogenic conversion. Under environmental stress, these prophages may induce lytic activation, releasing infectious viral particles that promote the horizontal transfer of ARGs [202,203]. Notably, phage–plasmids (P-Ps), as unique MGEs, combine the lytic propagation traits of phages with the autonomous replication ability of plasmids. Through high-frequency gene exchange and shared genetic pools, P-Ps significantly accelerate the cross-species spread of ARGs within bacterial communities [204]. It is important to emphasize that the role of phages extends beyond serving as passive gene-transfer vehicles. Recent studies reveal that phages preferentially lyse plasmid-free cells that have shed fitness costs, thereby alleviating the competitive disadvantage typically faced by plasmid-carrying strains. Even in the absence of HGT events, phages can effectively promote plasmid persistence through targeted lysis [205]. However, taxonomic perspectives indicate variations among phage species in their involvement in gene transfer: only a limited number of phage species have been shown to carry specific types of ARGs [206]. These differences may relate to host range, infection mechanisms, and environmental adaptability. A deeper understanding of these mechanisms will enhance our grasp of the pathways underlying bacterial resistance dissemination and support the development of more effective intervention strategies.

Compared to conjugation and transduction, transformation represents a more autonomous route of HGT. It is defined as the uptake and integration of exogenous, free DNA fragments from the surrounding environment by bacterial cells, a process that can occur naturally or be artificially induced. Multiple studies have demonstrated that the use of antimicrobial agents can substantially enhance the transformation efficiency of bacterial cells [207,208,209], providing a key mechanism for bacterial adaptation and the evolution of antibiotic resistance.

### 3.3. Transmission Pathways of ARGs

Globally, antibiotics are extensively used in clinical care, animal husbandry, and agriculture. However, the lack of stringent regulation and rational usage protocols has led to widespread and prolonged irrational use of antibiotics. Such misuse accelerates the dissemination of antibiotic resistance through multiple pathways.

Under the selective pressure of indiscriminate antibiotic use, ARB carrying ARGs are continuously selected and enriched in humans, animals, and their associated environments. These resistant bacteria can colonize sites such as the human and animal gut [210], making the intestinal microbiota an important reservoir and exchange hub for ARGs [211]. Studies confirm that the abundance and diversity of ARGs in the human gut microbiome are significantly correlated with the frequency of antibiotic use [212], often resulting in short or long term increases in intestinal resistome abundance [213].

Resistant bacteria colonizing hosts are primarily released into the external environment via excretion. For example, through fecal discharge, ARB and ARGs enter environmental compartments such as water and soil, enabling cross-boundary transmission from individuals to ecosystems. Once in environmental media, the subsequent fate and dissemination dynamics of ARGs and ARB constitute a critical link in the spread of AMR. Numerous studies have demonstrated the remarkable persistence of ARGs in aquatic systems, soils, and sediments [113,148]. This persistence can be attributed to the combined effects of several factors previously discussed: the continued survival of ARB under environmental stresses [172], and the active exchange of ARGs among environmental bacterial communities via HGT mechanisms [189]. Such durability transforms the environment into a long-term reservoir and reactor for the preservation, amplification, and spread of resistance determinants.

Beyond environmental discharge, ARB and ARGs can also spread directly within the biosphere through close contact. For example, humans and their companion animals can share ARB such as MRSA and ESBL-producing *Escherichia coli*, achieving cross-species transmission through direct contact [214]. Furthermore, evidence of horizontal transfer of ARGs (e.g., *bla*_TEM_) has been documented between wild birds and their habitats (water and soil) [215]. Notably, certain highly transmissible plasmids (such as the IncX3 type plasmid carrying *bla*_NDM-5_) further increase the risk of global spread of ARGs [86]. In the context of globalization, frequent international travel, trade, and human mobility have created conditions for the cross-regional spread of ARB and ARGs [216], enabling locally emerged resistance problems to rapidly escalate into broader regional or even global public health challenges. Catastrophic events such as armed conflicts, floods, and typhoons act as “perfect storms,” further exacerbating the transboundary transmission risks of antibiotic resistance. Armed conflicts, by triggering mass displacement, dismantling health systems, and aggravating pre-existing structural health disparities, create fertile breeding grounds for the emergence and spread of antibiotic-resistant bacteria [217,218,219]. Similarly, natural disasters—including floods, hurricanes, and earthquakes—disrupt water and sanitation infrastructure, induce sewage overflows, and displace populations, thereby facilitating the rapid dissemination of antibiotic-resistant bacteria and resistance genes across the environment–human interface [220,221]. These disasters not only directly undermine healthcare systems but also, through population movement and environmental contamination, transform localized resistance into urgent public health emergencies of international concern. Consequently, global efforts to combat antimicrobial resistance must integrate humanitarian crises and climate change as central considerations, establishing surveillance and rapid response mechanisms tailored to vulnerable regions.

**Figure 3:**
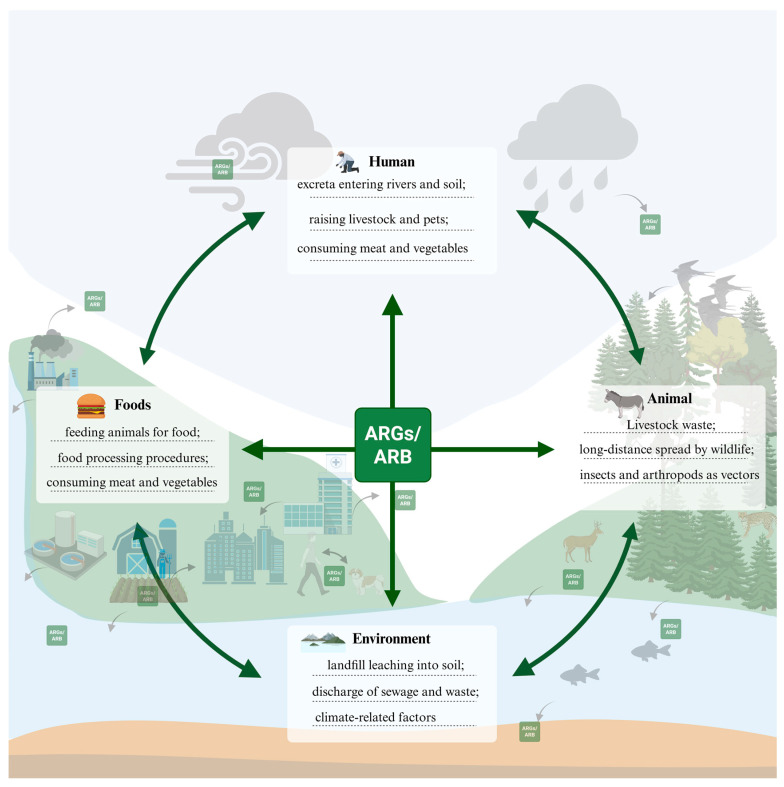
Transmission Pathways of ARGs in Four Key Ecosystems. Created in BioRender. lee, l. (2026) https://BioRender.com/24lgg5t (accessed on 8 March 2026).

Current evidence indicates that the dissemination of ARGs has transcended single ecological boundaries, forming a cross-media diffusion network (Figure 3). In natural aquatic environments, effluent discharges from wastewater treatment plants [143], antibiotic use in aquaculture [222], and surface runoff [223] collectively constitute major aquatic pathways for ARG spread. In soil ecosystems, long-term application of organic fertilizers containing resistant bacteria has turned agricultural fields into persistent reservoirs of ARGs [113], where MGEs (e.g., plasmids, integrons) significantly accelerate the horizontal transfer of resistance determinants. Of particular concern is the bidirectional exchange between “resistance hotspots” such as hospitals and livestock farms and the natural environment, creating a multidirectional cycling mechanism. This cycle endows ARGs with a near-ubiquitous ecological distribution. The environment, therefore, acts not merely as a backdrop for ARG exchange, but also as a source, vehicle, and ultimate sink of antibiotic misuse. The spread of ARGs is a multifactorial process, driven by anthropogenic activities across agricultural, industrial, and domestic sectors, and modulated by natural factors such as climate change. Together, these dynamics pose a profound and interconnected threat to global ecological security and human health, underscoring the imperative for integrated mitigation efforts under the One Health framework.

## 4. Conclusion and Perspectives

The evidence presented in this review confirms that AMR is an ecological phenomenon sustained by the continuous flow of ARB and ARGs among humans, animals, food systems, and the environment. These compartments do not operate in isolation; each serves simultaneously as a reservoir, a recipient, and a conduit for resistance determinants, largely driven by human activities across healthcare, agriculture, and waste management. Analysis of key transmission pathways—from clinical effluent and agricultural runoff to global food distribution networks—reveals an interconnected web of risks that facilitates the worldwide dissemination of ARGs.

Addressing this pervasive threat requires a strategic shift from compartmentalized approaches to genuinely integrated governance. Successfully mitigating the AMR crisis depends on translating the “One Health” principle from concept into coordinated, equitable, and sustained action across human, animal, environmental, and food system health. Moving forward, efforts must focus on implementing harmonized surveillance, aligning policies across sectors, and advancing research to quantify ARG transmission at key interfaces. Only through such inclusive and persistent collaboration can we effectively control the spread of resistance, protect human and animal health, ensure food safety, and maintain ecosystem integrity for future generations.

## Figures and Tables

**Table 1:** Distribution of important MDR bacteria across human, animal, food, and environmental niches.

Gram Stain	Important MDR Bacteria	Ecological Niche	References
Gram-negative	ESBL-Enterobacteriaceae (e.g., *E. coli*)	Human urinary tract infections (UTIs), bloodstream infections, and pneumonia, as well as food derived from both animal and plant sources.	[13,14,15,16,17]
	carbapenem-resistant Enterobacteriaceae (CRE)	Various niches within humans, animals, the food supply, and the environment.	[18,19,20,21,22,23,24,25,26]
	*Pseudomonas aeruginosa*	Infections in different parts of the human body (e.g., lungs and urinary tract), food products, farm animals, and aquatic environments.	[24,25,26,27,28]
	*Acinetobacter baumannii*	Infections in various human body sites (such as the lungs and urinary tract), food products, and aquatic environments.	[29,30]
Gram-positive	MRSA (Methicillin-resistant *Staphylococcus aureus*)	Infections in various human body sites (such as SSTIs), food-producing animals, and solid surfaces in diverse environmental settings.	[31,32,33,34]
	VRE (Vancomycin-resistant *Enterococci*)	Infections in various parts of humans and animals, the food chain, wildlife, and hospital settings.	[35,36,37,38]

**Table 2:** Drivers of antibiotic resistance gene dissemination and evolution.

Category	Drivers	
Evolutionary Drivers	Historical/Origin Drivers	Natural selection pressures
		Ecological competitive pressures
	Contemporary (Mechanistic) Drivers	Enzymatic inactivation or modification of antibiotics
		Structural alteration of drug targets or reduced target affinity
		Modulation of membrane permeability (efflux pump/porin)
		Mutations in core metabolic genes
		Horizontal gene transfer (HGT)
Transmission Drivers	Soil Environment	Mineral constituents (e.g., quartz, kaolinite, montmorillonite)
		Organic matter (e.g., humic acid, biochar, soot)
		Soil physicochemical properties (e.g., pH)
		Microplastic pollution
	Aquatic Ecosystems	Dissolved oxygen and nutrient concentrations
		Nanomaterials and heavy metals
		Oxidants
	Global Climate Change	Warming temperatures
		Extreme weather events (e.g., floods, heavy rainfall)
	Biological Vectors	Insects (e.g., Houseflies, Cockroaches)
		Wildlife (e.g., Wild Birds)

## Data Availability

No new data were created or analyzed in this study. Data sharing is not applicable.
